# Glycine Receptor Inhibition Differentially Affect Selected Neuronal Populations of the Developing Embryonic Cortex, as Evidenced by the Analysis of Spontaneous Calcium Oscillations

**DOI:** 10.3390/ijms21218013

**Published:** 2020-10-28

**Authors:** Denisse Ávila, Eduardo Aedo, Miguel Sánchez-Hechavarria, Claudio Ávila, Ariel Ávila

**Affiliations:** 1 Biomedical Sciences Research Laboratory, Department of Basic Sciences, Faculty of Medicine, Universidad Católica de la Santísima Concepción, 4090541 Concepción, Chile; davila2016@udec.cl (D.A.); edaedo@udec.cl (E.A.); misanchez@ucsc.cl (M.S.-H.); 2 School of Chemical and Process Engineering, University of Leeds, LS2 9JT Leeds, UK; C.R.Avila@leeds.ac.uk

**Keywords:** calcium, development, cortex, glycine receptor, Gini coefficient, PCA

## Abstract

The embryonic developing cerebral cortex is characterized by the presence of distinctive cell types such as progenitor pools, immature projection neurons and interneurons. Each of these cell types is diverse on itself, but they all take part of the developmental process responding to intrinsic and extrinsic cues that can affect their calcium oscillations. Importantly, calcium activity is crucial for controlling cellular events linked to cell cycle progression, cell fate determination, specification, cell positioning, morphological development and maturation. Therefore, in this work we measured calcium activity in control conditions and in response to neurotransmitter inhibition. Different data analysis methods were applied over the experimental measurements including statistical methods entropy and fractal calculations, and spectral and principal component analyses. We found that developing projection neurons are differentially affected by classic inhibitory neurotransmission as a cell type and at different places compared to migrating interneurons, which are also heterogeneous in their response to neurotransmitter inhibition. This reveals important insights into the developmental role of neurotransmitters and calcium oscillations in the forming brain cortex. Moreover, we present an improved analysis proposing a Gini coefficient-based inequality distribution and principal component analysis as mathematical tools for understanding the earliest patterns of brain activity.

## 1. Introduction

The cerebral cortex is where the higher order functions of the brain reside and thus, understanding its developmental process can help us to understand the etiology of important cognitive dysfunctions and mental disorders [1]. Projection neurons are born in the dorsal telencephalon, at the ventricular zone, and account for the majority of cortical neurons. After development, they will release glutamate and provide excitation to cortical circuits. Dysfunction on their development can cause serious brain abnormalities ranging from minor anatomical changes to marked micro or macrocephaly. These abnormalities can be linked to numerous mental disorders including epilepsy, learning disabilities and mental retardation [2]. Likewise, cortical interneurons have been found to be involved in epilepsy, autistic spectrum disorder and schizophrenia [3], among other mental illnesses. In particular, cortical interneurons are born at the ganglionic eminences, outside the cortex, and account for approximately 10% of all cortical neurons, providing inhibition and control to mature cortical circuits [2,4]. They characteristically migrate to the cortex adopting their final position integrating into cortical circuits alongside projection neurons [5].

Neurotransmitters and neurotransmitter receptors are mostly studied in the context of mature neuronal communications. However, these signaling molecules and receptors are also present in immature neurons and progenitor cells [6,7,8,9,10]. Glycine receptors (GlyR) are a representative example of this phenomenon [11,12,13] and have recently been linked to autism [14,15]. They are present in dorsal progenitors and migrator migratory interneurons, contributing to the control cell cycle dynamic [13], cell migration [16] and morphological development [17]. As a consequence, the lack of GlyR affects circuit development leading to enhanced susceptibility to epileptic compounds [17]. Importantly, application of glycine triggers in a calcium influx at the ventricular zone (VZ) of the developing cortex [13]. This is a similar response to the one elicited by GABA, and it correlates with the fact that both neurotransmitter systems affect the cell cycle dynamic in cortical progenitors [13,18,19]. Complementarily, GlyR inhibition depresses calcium activity in interneurons. In normal conditions, upon glycine receptor activation, interneurons are depolarized, activating voltage gated calcium channels leading to the entry of calcium, which can trigger downstream effects on the cellular behavior. As a result, inhibition of GlyR has been shown to block calcium spikes and impair neuronal migration [16]. This is again mirrored with the actions of GABA on interneurons [7]. However, due to the various types of GABA receptors, it has only been possible to precisely address the role of GlyR subunits on the control of cortex development [13,16,17]. Thus, along with providing insights on the role of GlyR, inhibition of GlyR is a useful strategy to understand the function of neurotransmitters during development.

During development, calcium oscillations are produced by the external inflow of calcium and by internal exchange of ions from organelles and the cytoplasm [20,21,22,23]. As such, they are not only a read out of maturing synaptic activity in the brain [24,25], but are also related to the cellular events taking place during neural development [26,27,28]. The earliest oscillations found on the developing cortex are characterized by low frequency and smaller amplitudes compared to oscillations found in the postnatal cerebral cortex [29]. At this place, calcium waves play a role in a variety of developmental processes such as the generation of neurons at progenitor regions [29,30], differentiation [31], migration, axonal growth, morphological development and maturation of electrophysiological properties [16,32]. Once brain structures are formed, cell survival of cortical neurons has also been shown to be affected by the modulation of activity [33,34]. Likewise, after birth, calcium oscillations fulfill additional roles through the control of gene expression and the general developmental process, which more and more is shaped by emerging synchronous activity [35]. Importantly, the same initial calcium oscillations are modulated or even driven by the activation of neurotransmitter systems. It has been reported that this type of calcium activity can participate in cell fate determination, cell proliferation [29,36], neurogenesis, cell migration [6,7,16], morphological maturation [37] and neuronal specification [28,38,39].

The preferred method of analysis of calcium oscillations has been based on defining and counting events, even after recognizing the marked differences between the first forms of calcium activity and later synchronized waves and spikes. This method has been applied with success in mature circuits, but its application to developing oscillations is not always feasible. Unbiased methods include linear techniques that determine average mean values, minimum and maximum values, and statistics variations, providing access only to limited qualitative information. On the other hand, power spectrum analysis is a calculation of the energy of the signal as a function of the frequencies present in the series (computed by transforming the signals to the frequency domain). This analysis is useful when individual events cannot be easily identified, and thus it can offer a better approach to evaluate calcium activity in developing neurons [39]. Likewise, principal component analysis (PCA) is a projection method searching for a low-dimensional representation of the data. This can be used to identify underlying variables affecting the system, the effects on spontaneous cell activity, and the presence of clusters and trends, from multidimensional data sets such as those from the calcium oscillation signals.

Here, we analyzed calcium activity on developing excitatory and inhibitory neurons of the cortex in control conditions and in response to inhibition of glycine receptors to further understand the actions of neurotransmitters and calcium oscillations during development of the brain. Moreover, we present the use of unbiased methods for the analysis of calcium oscillations applying a Gini coefficient-based inequality distribution, power spectrum analysis, and principal component analysis as a way of finding hidden information in the calcium oscillations of immature neuronal cells.

## 2. Results

### 2.1. Spontaneous Calcium Activity in Developing Cortical Projection Neurons Is Differentially Affected along the Cortical Wall by the Inhibition of Glycinergic Neurotransmission

**Figure 1:**
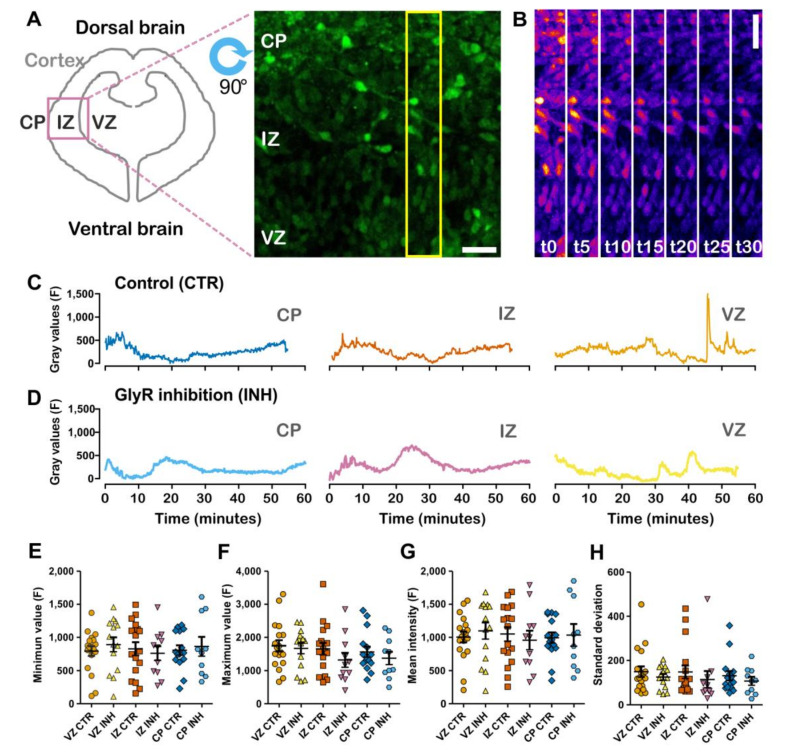
Calcium imaging and general analysis of oscillations in developing cortical projection neurons. (**A**) Scheme depicting a coronal section of the developing embryonic brain at E13 and representative image of Fluo4 labeled cortical projection neurons. VZ: Ventricular zone; IZ: Intermediate zone; CP: Cortical plate. Yellow rectangle shows selection expanded in B. Scale bar = 28 μm. (**B**) Pseudo-colored Fluo4 calcium signal. t in minutes. Scale bar = 28 μm. (**C**,**D**) Control and under inhibition sample traces for each zone of the cortex. Blue, CP control condition; Orange, IZ control condition; Light orange, VZ control condition; Light blue, CP under inhibition; Rose, IZ under inhibition; Yellow, VZ under inhibition. (**E**,**F**) Minimum and maximum values of intensity along the time course of the recording. (**G**) Mean intensity. (**H**) Standard deviation of intensity measurements. *N*: 18 (VZ CTR), 14 (VZ INH), 17 (IZ CTR), 11 (IZ INH), 15 (CP CTR) and 10 (CP INH).

**Figure 2:**
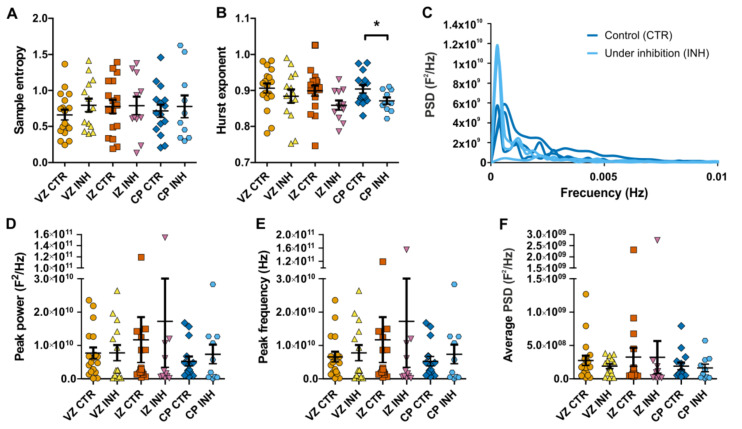
Entropy, index of dependence and power spectrum analysis of calcium oscillations in developing cortical projection neurons. (**A**) Sample entropy in control and under inhibition along cortical zones. (**B**) Index of dependence (Hurst exponent). (**C**) Sample grouped power spectral density (PSD) of ventricular zone cells. Control condition is shown in blue and GlyR inhibition condition in light blue traces. (**D**,**E**) Peak power and peak frequency of power spectra. (**F**) Average PSD. *N*: 18 (VZ CTR), 14 (VZ INH), 17 (IZ CTR), 11 (IZ INH), 15 (CP CTR) and 10 (CP INH). * *p* < 0.05.

Embryonic cortical projection neurons respond to the application of glycine and GABA with an increase in intracellular calcium [11]. For glycine, this has been shown to be most evident in the outer layers of the cortical wall at E17 [11]. Now, we found a similar pattern of activation at E13 (Supplementary video S1), where the response is most evident in the cortical plate, but is also present in progenitor regions, as shown before [13]. Therefore, we wondered if spontaneous activity at this early stage could be selectively influenced by neurotransmitters and used the inhibition of GlyR by strychnine as a proxy to understand the actions of these molecules at this stage. Calcium signal was measured at the ventricular zone (VZ), intermediate zone (IZ) and cortical plate (CP) regions, tracking Fluo4 fluorescent intensity on time and on individual developing projection neurons (Figure 1A,B). Calcium oscillations at the different zones were characterized by slow waves and sparse bursts of activity in tested experimental conditions and across cortical regions (Figure 1C,D). In a range up to 4095 gray values, non-normalized signals fluctuated on average from 800 to 1800, with a mean intensity value around 1000 (Figure 1E–H). While entropy across conditions and cortical zones was unchanged, meaning that there was similar predictability along the series (Figure 2A), the estimated Hurst exponent was decreased on the cortical plate upon GlyR inhibition (Figure 2B). This later result indicates that inhibition of GlyR, at the upper layers of the developing cortex, is translated into a lower persistency in the calcium signal in this region. In addition, we performed a power spectrum analysis of the acquired signals and, although grouped power spectra seemed to differ at the ventricular zone (Figure 2C), average peak power, peak frequency and power spectral density (PSD) did not vary across regions and conditions (Figure 2D–F).

### 2.2. Migratory Interneurons Display Irregular Spontaneous Calcium Oscillations That Fluctuate in a Narrower Range upon Glycine Receptor Blockage

**Figure 3:**
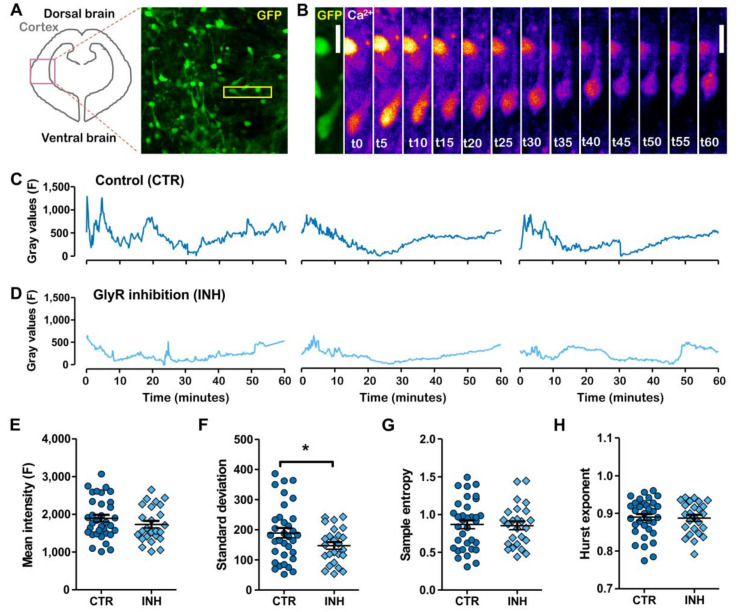
Calcium imaging and analysis of calcium oscillations in cortical interneurons. (**A**) Scheme depicting a coronal section of the developing embryonic brain at E13. Representative image of EGFP labeled interneurons entering the cortex from the bottom ventral brain to the top. The yellow rectangle shows the selection expanded in B. (**B**) Time course of pseudo-colored Fluo4 calcium signal. t in minutes. Scale bar = 14 μm. (**C**) Control sample traces. (**D**) Strychnine treated condition sample traces (INH). (**E**) Mean values of intensity along time course. (**F**) Standard deviation of intensities along time course of the experiment. (**G**) Sample entropy calculation result. (**H**) Hurst exponents. *N*: 34 (CTR), 26 (INH). * *p* < 0.05.

It has been shown that interneuron cell migration and calcium activity are impaired by inhibition of glycinergic neurotransmission [16]. Thus, we re-examined calcium oscillations recorded on individualized migratory interneurons, aiming at uncovering hidden effects of GlyR inhibition on this spontaneous activity. Migratory cortical interneurons were identified by their expression of GFP and the Fluo4 signal was tracked on time and space measuring intensity fluctuations on moving cells (Figure 3A,B). Here, oscillations were also characterized by slow waves and a sparse burst of activity (Figure 3C,D). Mean, minimum and maximum values of non-normalized measurements were unchanged between conditions, while standard deviation was decreased upon GlyR inhibition (Figure 3E,F). In this case, sample entropy and Hurst exponent calculations did not show differences between conditions (Figure 3G,H). Therefore, we hypothesized that a more in depth analysis of the power spectrum could better describe the effects of blocking neurotransmission on the developing brain.

### 2.3. Power Spectral Analysis of Calcium Oscillations Reveals New Insights on the Effect of Neurotransmitter Receptor Inhibition in Interneurons

**Figure 4:**
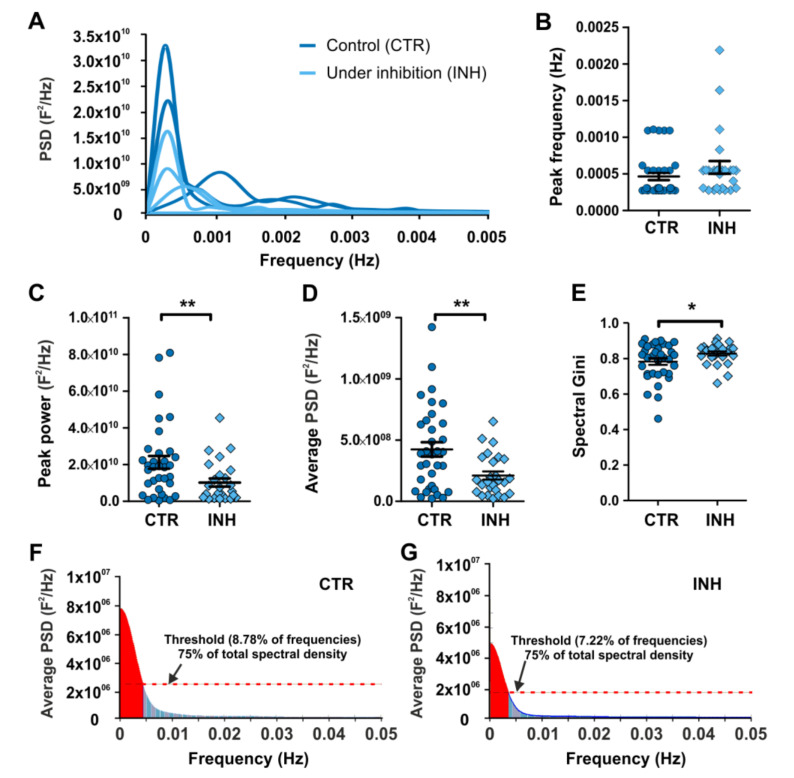
Power spectrum analysis of calcium oscillations in cortical interneurons. (**A**) PSD of grouped data. Control condition is shown in blue and GlyR inhibition condition in light blue traces. (**B**) Peak frequency. (**C**) Percentage of the spectrum covered by the peak power. (**D**) Average PSD. (**E**) Spectral Gini calculated for control and under inhibition power spectra. (**F**,**G**) Global average PSD of experimental conditions. *N*: 34 (CTR), 26 (INH). * *p* < 0.05, ** *p* < 0.01.

The power spectrum analysis showed that calcium oscillations, present in migratory interneurons, are characterized by low frequency oscillations concentrated in a rage below 0.05 Hz (Figure 4A). In control conditions, there was a predominant frequency around 0.00027 Hz and additional peaks in the range of 0.001–0.003 Hz. In contrast, upon GlyR inhibition, there was still a predominant frequency around 0.00027 Hz, but this was comparatively smaller and secondary components were nearly absent (Figure 4A). Interestingly, while peak frequency was unchanged, peak power and average PSD decreased upon GlyR inhibition (Figure 4B–D). 

### 2.4. Gini Coefficient-Based Inequality Calculation of Power Spectrum Pinpoint Differences Accounting for the Effect of Interfering with Calcium Oscillations in Migrating Interneurons

Considering the seemingly differential contribution of frequencies to the oscillations present in our experimental conditions, we assessed the use of the Gini coefficient to evaluate the differential distribution of frequencies in the calculated power spectra. The Gini coefficient became known for its application in economics to measure population income inequality. In the context of this study, when one parameter concentrates all the variability observed, the inequality is maximal and the calculated Gini coefficient equals 1. On the other hand, when the variability is equally distributed among all the parameters, then the equality is maximal and the Gini coefficient becomes 0. Consequently, applying this index to the power spectra of calcium oscillations serves as an index of the distribution of the frequencies present in the calcium traces. As such, we found that inhibition of GlyR caused an increase in the spectral Gini coefficient, when compared to the control condition. This was observed in the calculated average values and the accumulative distribution indicating a more homogeneous distribution of the energy of oscillations (Figure 4E–G). Concurrently, comparison between average power spectra shows that the percentage outside the 75% of the spectrum was decreased from 8.78% to 7.22% upon GlyR inhibition (Figure 4F–G).

### 2.5. Principal a Reveals Clustering by Cell Type and Treatment Effect on Cortical Neurons

**Figure 5:**
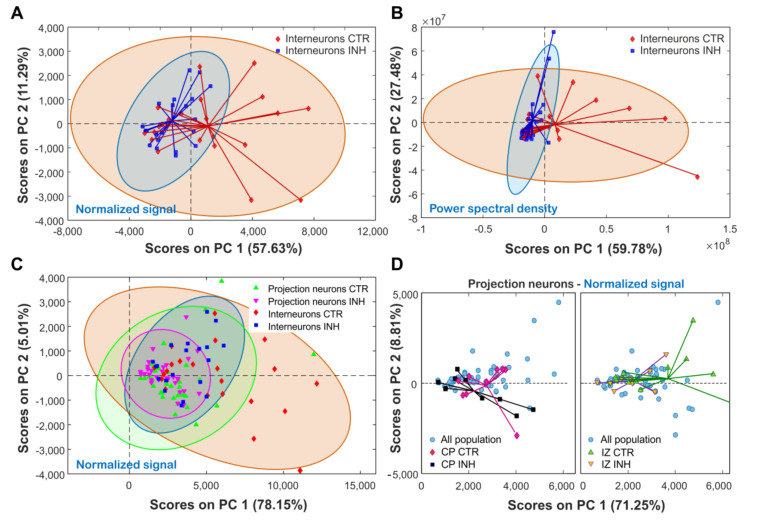
Principal component analysis (PCA) of spontaneous calcium oscillations. All graphs were produced by examining the first three components. (**A**) Treatment effect on normalized signal recorded on interneurons. Each point corresponds to a single sample signal, with both groups enclosed by a 95% confidence ellipse. (**B**) Treatment effect on PSD calculated on interneurons. (**C**) Treatment effect on normalized signal on projection neurons and interneurons. (**D**) Treatment effect on CP and IZ projection neurons, with individual groups plotted against the whole projection neuron sample population.

A principal component analysis was carried out using the normalized (baseline corrected) raw signals (Figure 5A), aiming to explore alternative methods to differentiate the cellular activity. Results show clustering of the data, clearly differentiating between CTR and INH groups. A three component model was able to explain 77% of the variance, with principal component 1 (PC1) capturing 57%. As a comparison, the same analysis was performed over the PSD signal, with a three component model capturing 93% of the variance (Figure 5B). In both cases data was mean centered, which positioned the points with less variation to the bottom left of the scatter plot. For CTR interneurons, signals with higher activity showed larger fluctuation along PC1 and PC2, while higher activity of INH interneurons signals was primarily observed along the PC2 axis. In addition, a spider net was used to visualize the central spot of the groups (equivalent to the center of mass for the cloud of points). The relative location of this central point can be used as a characteristic feature to differentiate between groups, and to assess the impact of each of the components in the average sample. Thus, regardless of the used input data, we observed that the center of mass was displaced along PC1 in response to GlyR inhibition on interneurons.

Relative to interneurons, projection neurons showed a narrower variability range when comparing both using a PCA model with three components (Figure 5C), with a 95% confidence ellipse as reference (no filtering applied). Moreover, the same shift observed between CTR and INH for interneurons was observed for the individual projection neurons groups. Figure 5D shows two of these groups contrasted by the total sample population (projection neurons only). Even these individual groups overlapped and were too close to allow significant differentiation, in all the cases CTR projection neurons showed a shift along PC1 relative to their INH counterpart.

## 3. Discussion

The work presented here analyzed the earliest patterns of brain activity in the form of calcium oscillations in the embryonic developing cerebral cortex. We focused our analysis on dorsal progenitors and projection neurons and compared their activity patterns to cortical interneurons in control conditions and under the inhibition of a neurotransmitter system. This allowed us to obtain a general overview of the action of glycinergic activation across cell types and regions in the developing cortex and to gain insights on how spontaneous calcium activity is structured early on during cortex development.

The actions of neurotransmitters during corticogenesis are linked to the modulation of calcium homeostasis. In particular, application of glycine to brain slices triggered widespread increase in intracellular calcium, particularly in the outer regions of the cortex at E13 [13] and E17 [11]. Application of GABA to the same preparation, triggered a similar and more homogeneous response throughout the cortex [11]. These findings are in agreement with the results reported by this study regarding the selective effect of strychnine on spontaneous activity at the cortical plate. It was found that immature neurons at the CP were the only ones among projection neurons responding to GlyR inhibition. This result contrasts with the fact that GlyR have been shown to affect cell cycle progression mainly at the VZ. Thus, it is possible that spontaneous calcium oscillations are not part of the mechanism downstream GlyR activation controlling cell cycle progression. Instead, spontaneous calcium activity might be relevant for the actions of GlyR in controlling morphological maturation, as this process began to take place in the CP at the recorded age. If that is the case, this result supports the idea that long term defects associated to the lack of GlyR might be due to the interference with individual cellular processes at different time points rather than arising as a consequence of the earliest defect.

Spontaneous calcium activity is a hallmark of developing neural systems. This activity is generally manifested as short spikes and long oscillations, with the latter being predominant in earlier stages of development. In this study, spontaneous calcium activity was measured in the developing cortex shortly after the beginning of corticogenesis in mouse embryos. At E13, it was found that spontaneous activity was characterized by slow wave oscillations with sparse bursts of activity. Interestingly, mixed calcium activity in projection neurons and migrating interneurons resembled activity recorded in growth cones, although with fewer bursts and long background oscillations [20]. At this age, activity coupled between neighboring cells was also observed, as it has been found at the VZ in posterior days during embryonic development [29,40]. However, it was also noted that the majority of cells displayed independent calcium oscillations.

Beyond its neurodevelopmental role, calcium signaling is involved in a myriad of cellular processes in which its role continues to be studied [23,36,41]. Thus, it is important to have robust methods of analysis that allow us to understand the underling biological mechanisms of calcium changes. Common methods of analysis for calcium oscillation have normally involved the visual or automatic identification of events based on criteria, detection thresholds or kinetic [39]. These methods work well in a number of settings and might be a precise readout of mature circuit activity. However, slow calcium oscillations, characteristically present in developing systems, are not amenable to the same analysis. Instead, developing calcium transients often do not have an easily recognizable regular shape. Therefore, here we applied unbiased methods of analysis searching to better assess the underlying biological phenomenon. Linear methods, based on statistical parameters, are widely used in neuroscience. They are very robust and are preferred because they accurately describe data sets and simplify the comparison between experimental conditions. In this study, the mean value, minimum and maximum values and standard deviation used as descriptors of the time series acquired in control and under inhibition were compared. However, these calculations provided limited information on the effects of GlyR inhibition, suggesting only changes in the variability of intensity values on interneurons. On the other hand, sample entropy calculation of time series exclusively showed differences in CP neurons in response to GlyR inhibition. In the same way, power spectrum and later analyses were selective in showing effects on interneurons. Thus, it would be interesting to study if these selective outcomes depending on the method of analysis relate to different underlying biological mechanisms.

With regards to the methodological approach used, power spectrum analysis appears as a reliable unbiased method of analysis for slow calcium oscillations occurring during brain development. Moreover, the ability to divide the spectrum opens up the possibility to selectively analyze the energy contribution of a subset of frequencies, which may account for different underlying cellular processes. Building on this, we proposed a Gini coefficient-based inequality distribution analysis for the better analysis and understanding of calcium activity on developing systems. In our study, we found increased Gini coefficient in inhibited neurons, implying that oscillations became concentrated around certain frequencies, changing to become more unequally distributed along the frequency range. This indicator makes it possible to obtain information, which is not reported by the traditional methods of spectral analysis, revealing previously unexplored elements about the nature of the distribution of biological oscillations. In consequence, now we do not only know that glycine receptor inhibition depressed calcium activity, but we also know that some activity might be differentially affected. This is an important advance, towards further dissecting downstream molecular mechanisms as it potentially provides an analysis tool to link experimental manipulations to cellular outcomes. The application of the Gini coefficient to biological phenomena has recently being explored. Applications of this coefficient include the analysis of electrocardiogram (ECG) signals [42], electroencephalogram (EEG) traces [43], functional magnetic resonance imaging [44] and gene expression analysis [45]. Interestingly, the use of the Gini coefficient for the analysis of ECG signal have allowed it to unveil the relationship between ECG changes and psychological stress [42]. This is remarkable as it contributes to obtaining a correlated readout of electrical activity and a cognitive process. Additionally, in assessment of fetal brain development employing postmortem diffusion magnetic resonance imaging, the Gini coefficient can be a simple, intuitive parameter for modeling fetal brain development because it correlates significantly with real fetal brain age [44].

Finally, PCA has been successfully applied to RNAseq data to define the population of cortical neurons [46,47,48]. Applied now to calcium oscillations we also were able to dissect cellular heterogenicity based on imaging data. Using power spectrum data instead of raw signal values captured a higher amount of variability. This increase in the model accuracy can be explained by the enrichment of useful information obtained from the PSD calculation. However, this increase was at the expense of losing half of the data points per signal (when implementing FFT), which could also contain important information. In physical terms, each of the principal components presented represents the influence of a single hidden variable (or a group of variables combined on a single parameter) that influenced cellular behavior. For the four models studied, PC1 captured around 60–75% of the variance, while PC2 capturing between 5 and 25% of it. From our analysis, a tree component model can be enough to generate a good insight into the process. Consequently, the dimensionality reduction obtained from the PCA analysis provided a qualitative method to identify these patterns, and remains a challenge to transform these observations into a quantitative estimate linked to a specific variable.

## 4. Material and Methods

### 4.1. Image Intensity Data Collection

Images analyzed in this study were obtained from data sets collected in a previous study, where calcium oscillations were partially analyzed on interneurons exclusively with the power spectrum [16]. More in detail, brain slices were obtained from Dlx5,6:Cre-IRES-EGFP transgenic mice, expressing EGFP on migrating cortical interneurons, at embryonic day 13 (E13). Slices were placed on holding inserts and allowed to recover on an air liquid interphase with neurobasal medium supplemented with N2, B27, penicillin-streptomycin and L-glutamine (Life Technologies, Gent, Belgium) at 37 °C for at least 1 hour. Loading of slices was done with Fluo4 AM at 37 °C, with agitation, for 30 minutes without holding insert. GlyR inhibition was achieved using strychnine at a final concentration of 1 μM, introducing it just before imaging. Recording of spontaneous activity was best performed using culture media on the air liquid interface at 37 °C and 5% CO_2_. Image acquisition was carried out using a Zeiss 200M inverted microscope fitted with an incubator chamber, coupled to an LSM510M confocal scanner head (Zeiss, Zaventem, Belgium) and connected to a MaiTai Titanium-Sapphire laser (Spectra physics, Irvine, CA, USA), for two-photon imaging. Fluo-4 was selectively excited using 820 nm pulsed light with a sampling frequency around 0.1 Hz. EGFP signal of interneurons was identified independently exciting at 900 nm wavelength. Additional details are described in the original article published by Avila et al [16]. As per the image analysis, fluorescence intensity measurements were performed using Imagej software V1.49k. Regions of interest (ROI) were defined to circle the entire cytoplasm of the cell and were repositioned in every frame using the ROI manager tool ensuring precise tracking of the cell along the entire time series. Then, mean intensity values were extracted measuring the circled area using the same tool.

### 4.2. Statistical Analysis

Average and standard deviation values for each time series were calculated using Prism software V5. Likewise, unequal variance *t*-test was used routinely to compare between control and strychnine-mediated GlyR inhibition.

### 4.3. Sample Entropy and Hurst Exponent Calculation 

Sample entropy calculation was applied to calcium imaging raw data, as shown previously [49]. Hurst exponent analysis was computed with a rescaled range (R/S), as shown before [39].

### 4.4. Power spectral Analysis

Power spectrum analysis was performed grouping data in subsets acquired at the exactly same frequency, transforming the signal to the frequency domain, and subsequently calculating power spectrum and power spectral density (PSD) by using the generic function available in LabView [50].

Due to the high laser intensity and repeated excitation needed to image calcium oscillation in time, the mean fluorescence intensity drift from the starting point decreasing in a time dependent manner. To correct for this physical effect and to prevent this factor from influencing power spectrum analysis, baseline was corrected offline as performed elsewhere using Octave software 2019 version 5.1.0 (https://www.gnu.org/software/octave/download.html), implementing a linear fitting in Labview (NI, Austin, TX, USA).

To compute the average spectra, a total of 2048 samples were subjected to computation through the Welch modified periodogram (Welch 1967) with a Hann window, using segments of 512 samples and overlapping periods of 256 samples.

### 4.5. Spectral Gini Coefficient Calculation

The Gini coefficient was calculated using the following equation:

$$G\left(x\right)=\frac{\sum_{i=1}^{N}\sum_{j=1}^{N}\left|x_{i}-x_{j}\right|}{2N\sum_{i=1}^{N}x_{i}}$$

where the income level of the *i*th [*i* = 1, 2. . . *N*] house is *xi* [42]. This equation has been adapted to evaluate the inequality in the powers of the electroencephalogram and electrocardiogram spectral components. For this purpose, each frequency of the power spectrum is considered as an individual house (H, highest frequency; L, lowest frequency) and the power of the corresponding frequency (X(f)) is considered as the house income. This allows one to quantify the spectral inequality in terms of the Gini coefficient and defines the spectral Gini coefficient (SpG) as it follows:

$$SpG_{f_{L}-f_{H}Hz}=\frac{\sum_{i=1}^{N}\sum_{j=1}^{N}\left|X\left(f_{i}\right)-X\left(f_{j}\right)\right|}{2\left(H-L+1\right)\sum_{i=L}^{H}X\left(f_{i}\right)}$$

where (*fL* − *fH* Hz) is the range of interest. Therefore, this equation was applied to the power spectra generated from calcium oscillations recorded on migratory interneurons.

### 4.6. Principal Component Analysis (PCA)

Exploratory PCA was performed using PLS_Toolbox V 8.5.2 (Eigenvector Research INC, USA) for use with MATLAB 8.5 (R2015a). All samples with a sampling frequency of 10 seconds per reading (0.1 Hz) were used for analysis. From these, signals were reduced to a fixed length (software requirement) containing the first 181 points, equivalent to 30 minutes of recording. For all the groups studied, the first three principal components were obtained from the raw signal (baseline corrected), and from their corresponding PSD calculation (for comparison). For Figure 5A,B data was mean centered with no additional filtering. For Figure 5C,D, only raw data was used without pretreatment.

## Supplementary Materials

Supplementary materials can be found at https://www.mdpi.com/1422-0067/21/21/8013/s1.
